# Outcomes of pars plana vitrectomy in the management and diagnosis of patients with infectious, non-infectious, and unidentified uveitis

**DOI:** 10.1007/s00417-024-06407-y

**Published:** 2024-02-16

**Authors:** Hande Celiker, Furkan Çam, Berru Yargı Özkoçak

**Affiliations:** 1 Department of Ophthalmology, Marmara University School of Medicine, Istanbul, Turkey; 2 Department of Ophthalmology, Marmara University Pendik Education and Research Hospital, Pendik, Istanbul, Turkey; 3 Beyoglu Eye Training and Research Hospital, University of Health Sciences, Istanbul, Turkey

**Keywords:** Endogenous endophthalmitis, Pars plana vitrectomy, Uveitis, Visual outcomes

## Abstract

**Purpose:**

To present the outcomes of pars plana vitrectomy (PPV) in patients with infectious, non-infectious, and unidentified uveitis, focusing on visual and clinical outcomes, diagnostic yield, and surgery-related complications.

**Methods:**

This retrospective, single-center study included patients who underwent 23-gauge PPV for the management of uveitis and had at least 6 months of follow-up. Patients were divided into infectious, non-infectious, and unidentified uveitis groups based on definitive diagnosis after surgery. Etiologies of uveitis, indications for surgery, diagnostic yield, visual outcomes, presence of cystoid macular edema (CME), immunosuppressive drugs, intraoperative and postoperative complications, and repeated vitrectomies were reviewed.

**Results:**

This study included 62 eyes of 54 patients. Twenty eyes were diagnosed with infectious uveitis, 24 eyes with non-infectious uveitis, and 18 eyes with unidentified uveitis. The diagnostic yield of vitrectomy was 41.7%. Mean BCVA significantly improved at postoperative 1 month compared to baseline and remained stable at following time-points in all groups. The most common early postoperative complication was increased intraocular pressure (17%), and late complication was cataract (36%). Nine eyes underwent re-vitrectomy and the most common cause was retinal detachment with proliferative vitreoretinopathy (PVR).

**Conclusion:**

PPV seems to be effective in diagnosing cases of unknown origin, improving visual acuity, and reducing the need for systemic immunosuppressive drugs. PVR is the most serious complication with poor prognosis that requires repeated surgery in patients with uveitis.
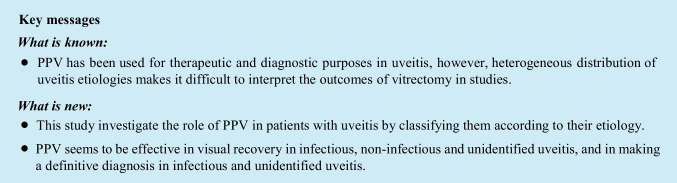

**Supplementary Information:**

The online version contains supplementary material available at 10.1007/s00417-024-06407-y.

## Introduction

Uveitis includes various disease entities and creates diagnostic and management challenges. The first step for successful management of uveitis is based on accurate diagnosis and appropriate treatment. Medical treatment remains the mainstay of treatment for uveitis [1]. However, failure of medical treatment or misdiagnosis of uveitis may cause permanent damage. Therefore, surgical interventions may be indicated for diagnostic sampling or to manage complications of uveitis [2–4].

Pars plana vitrectomy (PPV) has been used for the last four decades in the management of patients with uveitis [5] and advances in micro-incision vitrectomy surgery (MIVS) and new vitreoretinal instruments in recent years have made PPV prominent as a diagnostic and therapeutic tool in uveitis [2, 6]. The main benefits of PPV are the removal of membranes and vitreous opacities where infectious organisms and inflammatory mediators have accumulated [7, 8]. Studies have shown that performing PPV for diagnostic sampling or management of posterior segment complications also contributes to the improvement of inflammatory parameters such as postoperative visual acuity (VA), anterior and posterior segment inflammation scores, and cystoid macular edema (CME) [2, 3, 6, 7, 9]. Nevertheless, intraoperative and postoperative complications have limited the role of PPV in uveitis [2, 5, 10]. Surgery-induced inflammation, iatrogenic tears of inflamed retina during surgery, and postoperative anterior and posterior proliferative vitreoretinopathy (PVR), which is more common in uveitic eyes, are the major concerns about the outcomes of PPV in patients with uveitis [6, 10, 11].

The benefits of vitrectomy remain controversial due to the challenging nature of uveitis. In light of previous studies, we aimed to investigate the visual and clinical outcomes, diagnostic yield, and surgery-related complications of PPV in uveitis and compare the surgical outcomes between patients with different uveitis etiologies.

## Methods

### Study design and population

In this single-center retrospective study, we reviewed the clinical records of 62 eyes (54 patients) with uveitis who underwent 23-gauge PPV from January 2012 to March 2023 at our tertiary referral uveitis center. All patients had at least 6 months of follow-up after surgery. The study was approved by the local Institution Ethics Committee (No: 03.02.2023.312) and conducted in accordance with tenets of the Declaration of Helsinki. Indications for vitrectomy consisted of uncontrolled uveitis despite anti-inflammatory/anti-infectious treatment, acute sight-threatening uveitis with unknown origin, atypical presentation of uveitis, suspicion of intraocular malignancy, and complications of uveitis requiring surgical intervention. Patients with posttraumatic or postoperative endophthalmitis were excluded from this study. All patients underwent detailed ophthalmologic examinations including best-corrected visual acuity (BCVA), slit-lamp biomicroscopy, tonometry, fundus examination, and spectral-domain optical coherence tomography (SD-OCT). Ocular ultrasound, fluorescein angiography, and indocyanine green angiography were performed if required.

Diagnostic work-up was implemented on all patients to determine the etiology of uveitis at presentation. The preoperative diagnosis of infectious uveitis was initially based on clinical findings, serologic tests, and cultures of blood, urine, cerebrospinal fluid, or other body fluids. Eyes whose preliminary diagnosis could not be determined based on clinical findings and various diagnostic tests before surgery were included in the unidentified uveitis group. In patients with unidentified uveitis, suspected intraocular malignancy, or unknown origin of infectious uveitis such as endogenous endophthalmitis (EE) or viral retinitis, vitreous sampling was performed for diagnostic purposes at the beginning of PPV. Patients whose infectious origin was detected in the vitreous sample were included in the infectious uveitis group postoperatively. In addition, despite negative vitreous analysis, patients with positive microbiological cultures of any of the specimens (such as blood, urine, cerebrospinal fluid, and indwelling catheter), history of predisposing factors (such as diabetes, intravenous drug use, and malignancy), and peroperative highly suspected retinal findings were also included in the postoperative infectious uveitis group. Patients who underwent diagnostic PPV but whose all tests (vitreous analysis, serological test, microbiological culture) were negative were classified as unidentified uveitis. PPV was performed only for therapeutic purposes to patients with a definite etiology of uveitis before surgery. Hereby, the postoperative diagnosis of these patients was considered to be the same as their preoperative diagnosis.

The collected data from the patients’ medical records included the following: age, sex, laterality of eyes, duration between diagnosis and surgery, lens status, etiology of uveitis, anatomic localization of uveitis [12], systemic immunosuppressive drugs, BCVA, indications of vitrectomy, postoperative follow-up time, concomitant cataract surgery, additional surgical procedures, analysis of vitreous specimens, preferred tamponade agent, silicone oil (SO) removal time (if SO was used), intraoperative complications, early (≤2 weeks) and late (>2 weeks) postoperative complications, and repeated surgeries.

### Surgical technique

All patients underwent standard 3-port 23-gauge PPV (OS4 or Faros surgical platform; Oertli Instruments, Berneck, Switzerland) under local or general anesthesia by the same experienced surgeon (H.C.). In patients that underwent diagnostic PPV, vitreous sampling was performed primarily for diagnostic purposes. Procedures described in our previous study were performed for vitreous sampling [13]. Complete vitrectomy, removal of the posterior hyaloid membrane, and scleral indentation for vitreous base shaving were performed in all eyes. Other vitreoretinal procedures such as epiretinal membrane (ERM) or inflammatory membrane peeling, endolaser photocoagulation for retinal breaks, and gas, sterile air, or SO tamponade were performed as needed. Before intraocular tamponade injection, intravitreal anti-infectious agents (both vancomycin 1 mg/0.1 ml and ceftazidime 2.25 mg/0.1 ml for bacterial EE, amphotericin B 5 μg/0.1 ml for fungal EE) were administered in eyes with presumptive EE. Triamcinolone acetonide (4 mg/0.1 ml) was injected intraocularly if required.

### Outcomes

The main outcomes of this study were mean changes and percentage of improvement in BCVA (≤−0.3 logMAR) from preoperative to postoperative 1, 3, 6, and 12 months, diagnostic yield of vitreous specimens’ analyses, presence of CME on SD-OCT at baseline and postoperative 6 months, immunosuppressive drugs and doses prescribed at baseline and postoperative follow-up visits, additional surgical procedures, early (≤2 weeks) and late complications (>2 weeks), and causes of repeated surgeries. Patients were divided into three groups according to definitive diagnosis after vitrectomy as infectious uveitis, non-infectious uveitis, and unidentified uveitis groups. Mean BCVA at baseline and postoperative time-points and the presence of CME at postoperative 6 months were compared between these groups.

### Statistical analysis

The Statistical Package for the Social Sciences (SPSS) for Windows version 22.0 (IBM Crop., Armonk, NY, USA) was used for statistical analysis. The BCVA was assessed with an electronic Snellen’s chart (in European decimals) and converted into a logarithm of minimum angle of resolution (logMAR) for statistical analysis. Counting fingers, hand motion, and light perception were determined as 1.9, 2.3, and 2.7 logMAR, respectively. The distribution of the data was determined with histograms and Shapiro-Wilk test. Categorical data were presented as frequency (*n*) and percentage (%), and numerical variables as mean and standard deviation (SD) or median and interquartile range (IQR). Categorical variables were compared by using Fisher’s exact tests. We used the generalized estimation equation (GEE) method for the analysis of visual outcomes to account for the correlation between both eye data. A *p*-value < 0.05 was considered significant.

## Results

**Fig. 1:**
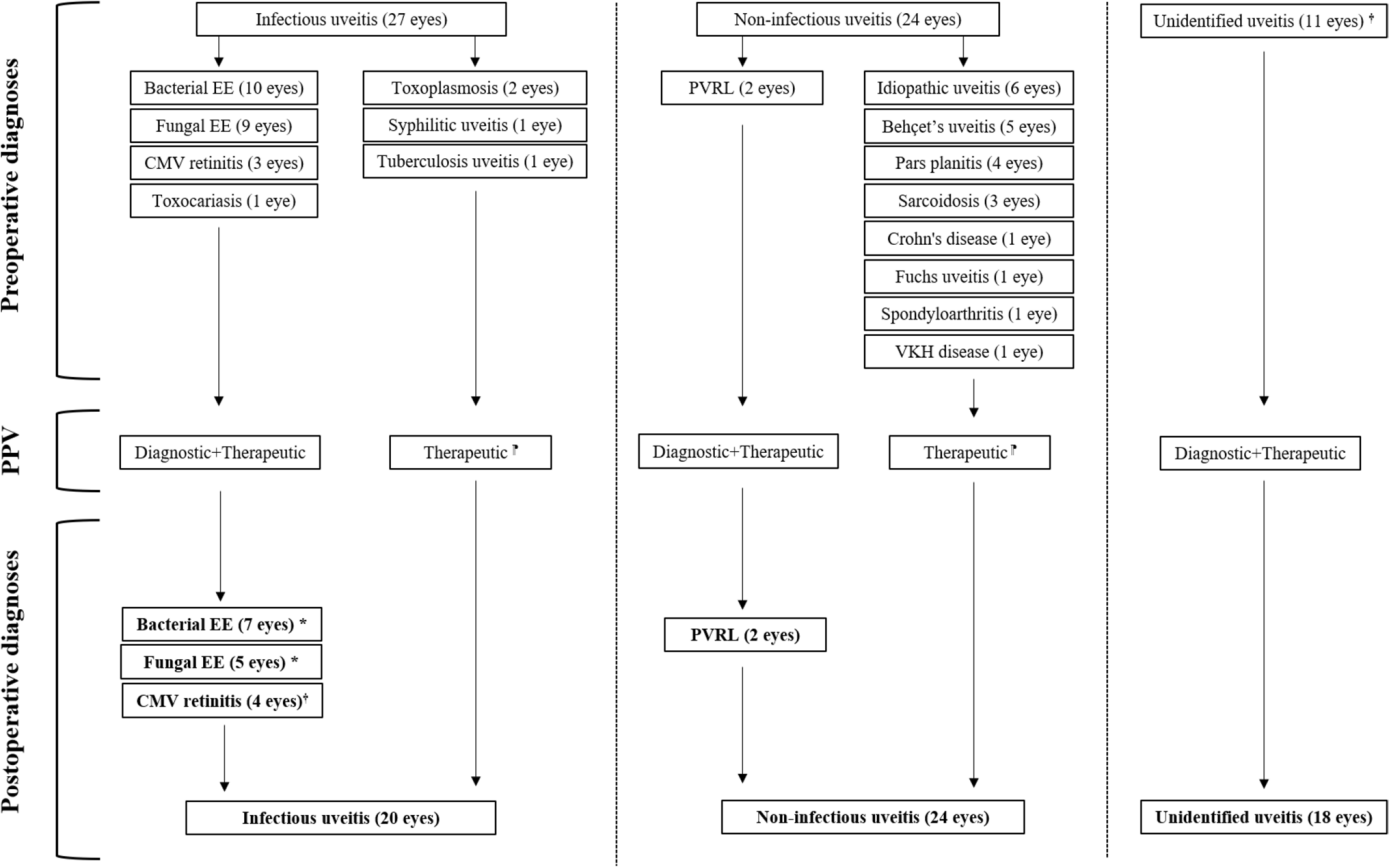
Preoperative and postoeprative diagnoses of eyes underwent pars plana vitrectomy. EE, endogenous endophthalmitis; PVRL, primary vitreoretinal lymphoma; CMV, cytomegalovirus; VKH, Vogt-Koyanagi-Harada; PPV, pars plana vitrectomy. *Two eyes with fungal EE and one eye with bacterial EE were still considered as infectious uveitis due to positive blood cultures, history of predisposing risk factors, and peroperative highly suspected retinal findings associated with EE. †CMV was detected in the vitreous analysis of one eye in the preoperative unidentified uveitis group. ⁋Postoperative and preoperative diagnoses of the eyes that underwent therapeutic PPV were the same

This study included 62 eyes of 54 patients, of whom 29 were men (53.7%). All patients had at least 6 months of follow-up. The mean age of the patients was 51.19±17.52 (range 5–82) years. The mean duration between diagnosis of uveitis and PPV was 27.47±41.52 months (median [IQR], 5.00 [43.25]), and mean follow-up time after surgery was 29.19±24.06 months (median [IQR], 23.50 [31.00]). The most common indications for surgery were vitreous opacity (*n*=38, 61.3%) and rhegmatogenous retinal detachment (*n*=7, 11%). Preoperative and postoperative diagnoses of the patients and the purposes of PPV are indicated in Fig. 1. Twenty-six eyes (42%) underwent PPV for therapeutic purposes and 36 eyes (58%) for both diagnostic and therapeutic purposes. Vitreous analysis was positive in 15 of these 36 eyes and the overall diagnostic yield of vitrectomy was 41.7%. Of the 15 eyes with positive vitreous analysis, five eyes were diagnosed with fungal EE, four eyes with bacterial EE, four eyes with cytomegalovirus (CMV) retinitis, and two eyes with primary vitreoretinal lymphoma (PVRL). The preoperative and postoperative diagnoses of eyes that underwent vitreous sampling are shown in Supplemental Table 1. In addition, three eyes with negative vitreous analysis (two eyes with fungal EE and one eye with bacterial EE) were postoperatively included in the infectious uveitis group due to positive blood cultures, predisposing risk factors (indwelling catheter and chronic systemic illness), and peroperative highly suspected retinal findings compatible with EE. In total, the postoperative diagnosis was non-infectious uveitis in 24 eyes (23 patients), infectious uveitis in 20 eyes (14 patients), and unidentified uveitis in 18 eyes (17 patients). The baseline demographics and clinical characteristics of patients are summarized in Table 1.

**Table 1:** Baseline demographics and clinical characteristics of all patients and infectious, non-infectious, and unidentified uveitis groups

Parameters	All patients	Infectious uveitis	Non-infectious uveitis	Unidentified uveitis
Number of patients/eyes	54/62	14/20	23/24	17/18
Age; mean±SD (min–max), age	51.19±17.52 (5–82)	53.79±20.88 (5–78)	49.52±14.41 (12–73)	51.29±19.17 (18–82)
Sex (male/female)	29/25	9/5	10/13	10/7
Laterality of eyes (right/left)	33/29	10/10	13/11	10/8
Unilateral PPV/bilateral PPV, *n*, patients	46/8	8/6	22/1	16/1
Follow-up period before PPV; months				
Mean±SD (min–max)	27.47±41.52 (0–240)	10.08±17.73 (0–57)	55.37±52.24 (1–240)	4.47±6.13 (0–25)
Median	5.00	1.00	45.50	3.00
Anatomical localization of uveitis, *n* (%), eyes				
Panuveitis	38 (61.3)	12 (60.0)	13 (54.2)	13 (72.2)
Posterior uveitis	18 (29.0)	8 (40.0)	5 (20.8)	5 (27.8)
Intermediate uveitis	4 (6.5)	-	4 (16.7)	-
Anterior uveitis (with dense vitritis)	2 (3.2)	-	2 (8.3)	-
Indications for surgery				
Vitreous opacity	38 (61.3)	12 (60.0)	11 (45.8)	15 (83.3)
Rhegmatogenous retinal detachment	7 (11.3)	5 (25.0)	2 (8.3)	-
Epiretinal membrane	6 (9.7)	2 (10.0)	4 (16.7)	-
Vitreous hemorrhage	4 (6.5)	-	3 (12.5)	1 (5.6)
Tractional retinal detachment	3 (4.8)	1 (5.0)	1 (4.2)	1 (5.6)
Intraocular lens dislocation	3 (4.8)	-	3 (12.5)	-
Subretinal abscess	1 (1.6)	-	-	1 (5.6)

*SD* standard deviation, *PPV* pars plana vitrectomy

Twenty-three eyes (37%) underwent PPV combined with phacoemulsification. ERM was performed in 29 eyes during the first surgery. Additionally, internal limiting membrane (ILM) peeling was done in five of these 29 eyes. Due to retinal inflammation, edema, fragility, and high risk of retinal hemorrhage, which are the nature of uveitic eyes, ILM peeling was postponed in some patients to be performed at the time of SO removal. Primary retinectomy was required in three eyes with PVR. Intraocular lens (IOL) was explanted in one eye with EE and left aphakic. Scleral fixated IOL was implanted in two eyes that underwent PPV due to IOL dislocation. Intraocular tamponade was performed in 50 eyes (SO, 38 eyes; gas [C3F8/SF6], nine eyes; sterile air, three eyes) at the end of the surgery. Of the eyes in which SO was preferred, 17 were infectious uveitis, seven were non-infectious uveitis, and 14 were unidentified uveitis. Of 38 eyes with SO, 27 eyes underwent SO removal during follow-ups. During SO removal, 10 eyes underwent ERM peeling, and two eyes combined phacoemulsification with ERM peeling. Furthermore, C3F8 gas was injected into four of these eyes at the end of the SO removal. Repeated vitrectomy and SO exchange were performed in six eyes with SO. It is planned to leave SO for a long time to prevent hypotonia or phthisis in three eyes (Supplemental Table 2).

**Fig. 2:**
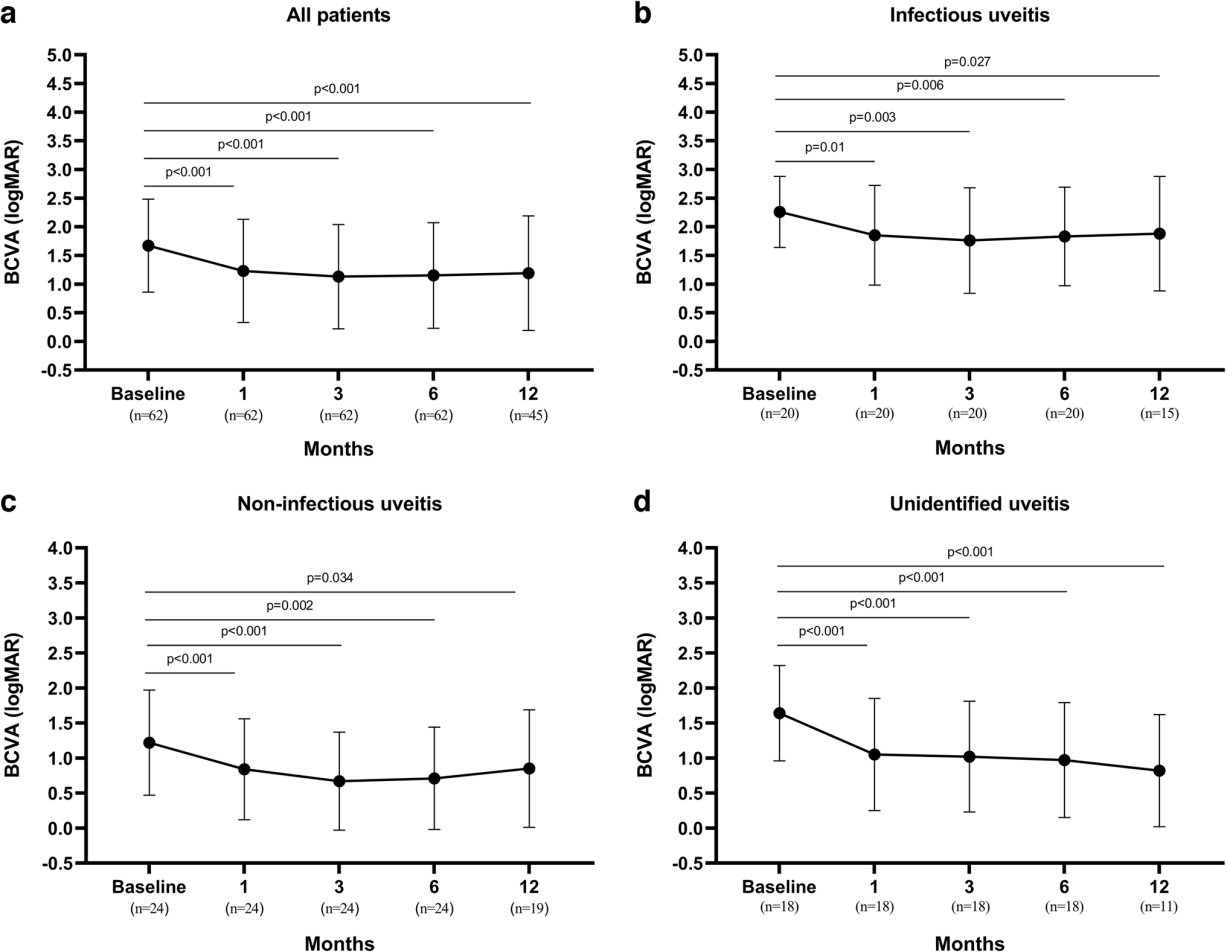
Changes in the mean logMAR best-corrected visual acuity (BCVA) from baseline to postoperative 1, 3, 6, and 12 months. BCVA was changed significantly after surgery compared to baseline in all patients (**a**), infectious uveitis (**b**), non-infectious uveitis (**c**), and unidentified uveitis (**d**). Statistical significance was determined using a generalized estimating equation (GEE) model

The mean logMAR BCVA in all patients and in each group at baseline and postoperatively 1, 3, 6, and 12 months are represented in Table 2. Visual acuity in the infectious uveitis group was significantly lower than both non-infectious uveitis and unidentified uveitis groups before surgery and postoperative follow-ups (Table 2). Mean logMAR BCVA significantly improved at postoperative 1 month compared to baseline and remained stable at subsequent postoperative time-points in all groups (Fig. 2).

**Table 2:** Preoperative and postoperative mean logMAR BCVA and presence of CME in patients with different uveitis etiologies

Parameters	All patients	Infectious uveitis (A)	Non-infectious uveitis (B)	Unidentified uveitis (C)	*p*-value*		
					A vs B	A vs C	B vs C
BCVA logMAR at baseline					**<0.001**	**0.003**	0.052
Mean±SD	1.67±0.81	2.26±0.62	1.22±0.75	1.64±0.68			
Median	1.90	2.30	1.00	1.60			
BCVA logMAR at 1st month							
Mean±SD	1.23±0.90	1.85±0.87	0.84±0.72	1.05±0.80	**<0.001**	**0.003**	0.464
Median	1.00	1.95	0.60	0.80			
Percentage of eyes with improvement in BCVA	48.4%	45.0%	41.7%	61.1%			
BCVA logMAR at 3rd month					**<0.001**	**0.007**	0.294
Mean±SD	1.13±0.91	1.76±0.92	0.67±0.70	1.02±0.79			
Median	0.70	1.90	0.40	0.70			
Percentage of eyes with improvement in BCVA	56.5%	45.0%	58.3%	66.7%			
BCVA logMAR at 6th month					**<0.001**	**0.002**	0.638
Mean±SD	1.15±0.92	1.83±0.86	0.71±0.73	0.97±0.82			
Median	0.70	1.90	0.50	0.70			
Percentage of eyes with improvement in BCVA	62.9%	55.0%	66.7%	66.7%			
BCVA logMAR at 12th month					**0.003**	**0.002**	0.692
Mean±SD	1.18±1.03	1.88±1.00	0.85±0.84	0.82±0.80			
Median	0.80	2.30	0.50	0.50			
Percentage of eyes with improvement in BCVA	57.9%	46.7%	57.9%	72.7%			
Presence of CME on SD-OCT, *n* (%)					***p*-value**^**†**^		
Preoperative							
Present	14 (22.6)	3 (15.0)	10 (41.7)	1 (5.6)			
Absent	25 (40.3)	5 (25.0)	11 (45.8)	9 (50.0)			
Undefined	23 (37.1)	12 (60.0)	3 (12.5)	8 (44.4)			
Postoperative 6th month							
Present	8 (12.9)	3 (15.0)	2 (8.3)	3 (16.7)			
Absent	54 (87.1)	17 (85.0)	22 (91.7)	15 (83.3)	0.646	1.000	0.636

*BCVA* best-corrected visual acuity, *logMAR* logarithm of the minimum angle of resolution, *SD* standard deviation, *CME* cystoid macular edema

*Statistical significance was determined using a generalized estimating equation model accounting for the correlation structure of paired eyes

^**†**^These *p*-values indicate the comparison of different groups regarding the presence of CME at the postoperative 6th month. Fisher’s exact test was used for statistical analysis

Before surgery, CME was present in 14 eyes (23%) and absent in 25 eyes (40%) on SD-OCT. On the other hand, SD-OCT images were unclear in 23 eyes (37%) due to dense vitreous haze at baseline. CME was detected in eight eyes (12%) at postoperatively 6 months and there was no significant difference between the groups in terms of the presence of CME (Table 2).

Systemic treatments of 16 patients (16 eyes) (16/54, 29%) who received immunosuppressive therapy preoperatively and postoperatively are summarized in Supplemental Table 3. Four of these patients were receiving oral corticosteroids, six were receiving immunomodulatory drugs, and five were receiving both oral corticosteroids and immunomodulatory drugs. Corticosteroids were completely cessated in all patients within the first 3 months after surgery and the number of immunosuppressive drugs used decreased in eight patients (8/16, 50%) until the last visit.

Intraoperative and postoperative complications of PPV are shown in Table 3. The most common surgery-related early postoperative complication was increased intraocular pressure (11 eyes; 17%) and the most common late postoperative complication was cataract (five of 13 eyes remained phakic after surgery, 38%). ERM was observed in seven eyes (11%) and ERM peeling was performed after SO extraction in these eyes. Postoperative refractory CME was developed in seven eyes (11%) and these eyes received intravitreal dexamethasone implant or anti-vascular endothelial growth factor injection for the management of CME. Repeated vitrectomy was indicated in a total of nine eyes (retinal detachment (RD), six eyes; VH, one eye; recurrent vitreous opacity, one eye; IOL dislocation into the vitreous, one eye) (Table 3). Three eyes required two or more re-vitrectomy due to RD with PVR. At the last follow-up, VA was worse than hand motion in five of nine eyes that underwent re-vitrectomy.

**Table 3:** Intraoperative and early and late postoperative surgical complications of patients

Parameters, *n* (%)		All patients (*n*=62)	Infectious uveitis (*n*=20)	Non-infectious uveitis (*n*=24)	Unidentified uveitis (*n*=18)
Intraoperative complications	Iatrogenic retinal break	3 (4.8)	2 (10.0)	1 (4.2)	-
Early postoperative complications (≤2 weeks)	Total	22 (35.5)	7 (35.0)	5 (20.9)	10 (55.5)
	Increased intraocular pressure	11 (17.7)	4 (20.0)	3 (12.5)	4 (22.2)
	Hypotonia	5 (8.1)	3 (15.0)	-	2 (11.1)
	Fibrin reaction	4 (6.4)	-	1 (4.2)	3 (16.7)
	Hyphema	2 (3.2)	-	1 (4.2)	1 (5.5)
Late postoperative complications (>2 weeks)	Total	28 (45.2)	10 (50.0)	11 (45.8)	7 (3.9)
	Retinal detachment with PVR	6 (9.7)	3 (15.0)	2 (8.3)	1 (5.5)
	Epiretinal membrane	7 (11.3)	2 (10.0)	2 (8.3)	3 (16.7)
	Cystoid macular edema	7 (11.3)	2 (10.0)	4 (16.7)	1 (5.5)
	Cataract*	4/11 (36.4)	1/4 (25.0)	2/4 (50.0)	1/3 (33.3)
	Phthisis	3 (4.8)	3 (15.0)	-	-
	Choroidal neovascularization	1 (1.6)	-	-	1 (5.5)
	Vitreous opacity	1 (1.6)	1 (5.0)	-	-
	Vitreous hemorrhage	1 (1.6)	-	-	1 (5.5)
	Glaucoma	2 (3.2)	-	1 (4.2)	1 (5.5)
	IOL dislocation into vitreous	1 (1.6)	-	1 (4.2)	-
Repeated vitrectomy	Total	9 (14.5)	4 (20.0)	3 (12.5)	2 (11.1)
	Retinal detachment with PVR	6 (9.7)	3 (15.0)	2 (8.3)	1 (5.5)
	Vitreous hemorrhage	1 (1.6)	-	-	1 (5.5)
	IOL dislocation into vitreous	1 (1.6)	-	1 (4.2)	-
	Vitreous opacity	1 (1.6)	1 (5.0)	-	-

*PVR* proliferative vitreoretinopathy, *IOL* intraocular lens

*Eleven eyes remained phakic after pars plana vitrectomy

## Discussion

The heterogeneous distribution of uveitis etiologies makes it difficult to interpret the outcomes of vitrectomy in studies. Therefore, in this study, we presented outcomes of PPV in our cohort with uveitis by grouping the patients into infectious, non-infectious, and unidentified uveitis according to etiologies of uveitis.

In several studies evaluating the effect of diagnostic PPV, the diagnostic yield varied from 14.3 to 61.5% [14–17]. A recent literature review of 16 studies reported a success rate of 44% for diagnostic PPV [9]. Diagnostic yields were compatible with the literature and a definitive diagnosis was achieved in 41.7% of these eyes. In addition, it has been observed that eyes with infectious uveitis and fungal EE are more easily diagnosed with vitreous sampling. These benefits of vitrectomy, along with the removal of infectious organisms from the vitreous cavity, emphasize its prominence in the definitive diagnosis and the management of infectious uveitis.

Changes in VA, the presence of CME, and the need for adjunctive medical treatment are among the most common reported outcomes after vitrectomy in patients with uveitis. Nearly all studies have reported varying degrees of improvement in VA after PPV for diagnostic or therapeutic purposes in patients with uveitis [2, 5, 7, 13, 14, 18, 19]. In a systematic literature review recently published by Henry et al. [2], improvement in postoperative VA was reported in 69% of eyes, unchanged in 18%, and worsening in 13% of eyes. Nearly the same results were reported in an earlier review [7]. Nevertheless, these studies included a limited number of patients with infectious. It is known that visual outcomes are generally poor in patients with infectious uveitis; nevertheless, favorable outcomes can be obtained with aggressive medical and surgical treatment [20]. In the presented study, eyes with infectious uveitis constitute approximately 30% of our study population and the mean BCVA in infectious uveitis before and after surgery was significantly lower than in non-infectious and unidentified uveitis groups. Nonetheless, similar to the aforementioned studies, the proportion of eyes with improvement in BCVA at the postoperative 6 months was 62%. In addition, mean BCVA improved significantly after surgery compared to baseline in all groups, including the infectious uveitis group. Therefore, these results confirm that PPV is effective in improving VA in patients with uveitis.

Removal of inflammatory mediators and infectious agents from vitreous and tractional adhesion on macula is thought to be effective in the elimination of posterior segment inflammation and resolution of CME [5, 6, 21, 22]. On the other hand, newly developed CME caused by PPV has also been reported in cases with uveitis [23]. While the effect of PPV on the resolution of CME was not clearly demonstrated in early studies investigating PPV for the management of uveitis, researchers reported a reduction in CME after surgery in recent studies [6, 23–25]. In a systematic literature review, preoperatively, 157 of 300 (52%) eyes had documented CME compared to 112 of 300 (37%) postoperatively [2]. In the presented study, we evaluated the presence of CME based on SD-OCT and therefore we consider that the presence of CME was lower. CME was observed in 14 eyes (22%) preoperatively, and CME resolved in 13 of these eyes after surgery. However, CME was detected in eight eyes (12%) of the eyes at postoperative 6 months and CME was not observed in seven of these eyes before surgery. SO was used in most of the eyes that developed postoperative CME. Although removal of inflammatory mediators from the vitreous with PPV provides resolution of CME, it has been suggested that some inflammatory mediators that increase after PPV disrupt the blood-aqueous barrier and cause fluid accumulation [26]. In addition, it is thought that SO may be responsible for ocular inflammation and lead to CME [27]. This surgery-induced inflammation and the presence of a risk factor such as systemic inflammation in uveitis patients may explain the development of postoperative CME.

Studies have reported inconsistent results regarding a change in the use of systemic immunosuppressive therapy after PPV [2]. Soheilian et al. [23] reported that 40.0% of patients needed fewer immunosuppressants to control postoperative inflammation following PPV. In another study, investigators showed that preoperative immunosuppressive therapy was discontinued in 44% and reduced in 17% of patients after surgery [25]. In the presented study, the number of drugs was reduced in 50% of the patients, and immunosuppressants were discontinued in 25% of these patients. Consistent with the literature, we noticed a significant rate of drug discontinuation and a considerably reduced need for immunosuppressive medications. It cannot be said that PPV is an alternative to systemic medical therapy for the treatment of uveitis. However, we may state that PPV is significantly beneficial in discontinuing immunosuppressive drugs and in avoiding complications secondary to these therapeutics.

Epiretinal and epiciliary membranectomy, 360° scleral indentation, vitreous base dissection, or intraocular tamponade injection with PPV is crucial in preventing common postoperative complications such as hypotonia, RD, or ERM [28–30]. The most frequently reported early complications are unusual intraocular pressure responses [10, 31]. Although we performed the requisite surgical procedures to avoid these complications, consistent with other studies, increased intraocular pressure and transient hypotonia developed most frequently in the early period of this study. Subsequently, cataract progression is the most common postoperative complication in nearly all studies and has been reported in up to 51% of cases [25]. In a literature review, the most common complications were determined as cataract (23.7%), glaucoma (16.7%), and ERM (15.3%), respectively [2]. Soheilian et al. [23] reported ERM formation in 23% of eyes and chronic inoperable RD in 6.7% of eyes after 25-gauge PPV. In parallel with these studies, we also found that cataract (36%) is the most common late postoperative complication. Postoperative PVR and RD have been reported infrequently for MIVS in cases with uveitis [10, 19, 25]. In a meta-analysis, Zhao et al. [9] showed that the need for second PPV was approximately 10% after diagnostic PPV. In another study, repeat PPV was performed in eight of 106 eyes (7.5%) that underwent 23- or 25-gauge PPV [10]. Svozilkova et al. [3] reported that 23 of 101 eyes (22.8%) underwent 20-gauge PPV were re-operated and most of them developed RD. In the presented study, postoperative RD with PVR developed in six eyes (9%) and re-vitrectomy was performed. This lower incidence compared to the study of Svozilkova et al. [3] could be explained by the use of 23-gauge PPV. On the other hand, this incidence seems less favorable than in studies with 25- or 27-gauge PPV [10, 31]. Consequently, surgery-related complications are major concerns related to intraocular surgery in inflamed eyes. We indicate that postoperative RD with PVR is the most serious complication with poor visual outcomes after PPV in uveitis.

The retrospective study design was the main limitation of this study. Various surgical indications and procedures due to the heterogeneous nature of uveitis are also among the limitations of this study such as the previous studies.

In conclusion, patients with uveitis are challenging group for vitreoretinal surgery due to both ocular and systemic inflammation. This study presented favorable outcomes in terms of diagnostic yield, improvement in VA, decrease in the presence of CME, and reduction in systemic medication required for inflammation control in eyes with uveitis that underwent PPV for various indications even in infectious uveitis. PPV may be an effective method in the management of uveitis in selected patients whose systemic inflammation can be controlled. Nevertheless, surgeons should be aware of retinal fragility, high risk of hemorrhage, and possible serious complications such as PVR in eyes with uveitis. Multicenter studies with larger groups are needed to demonstrate the benefit of PPV in patients with uveitis of extremely various (such as Vogt-Koyanagi-Harada disease, Behçet’s uveitis, Crohn’s disease, and ocular toxocariasis) and rare etiologies.

### Supplementary information

ESM 1 (PDF 441 kb)

ESM 2 (PDF 198 kb)

ESM 3 (PDF 757 kb)

## Data Availability

The data supporting the study findings are available from the corresponding author upon request.
