# Supplementary material for: Outcomes of pars plana vitrectomy in the management and diagnosis of patients with infectious, non-infectious, and unidentified uveitis
Source: Graefes Arch Clin Exp Ophthalmol. 2024 Feb 16;262(7):2237–46. doi: 10.1007/s00417-024-06407-y (PMC11222255; doi:10.1007/s00417-024-06407-y)
Supplement: Supplementary file 1 — (PDF 441 kb) [file 417_2024_6407_MOESM1_ESM.pdf]

Article title: Outcomes of Pars Plana Vitrectomy in the Management and Diagnosis of Patients with Infectious, Non-infectious, and Unidentified Uveitis

Journal name: Graefe's Archive for Clinical and Experimental Ophthalmology

Authors:

Hande Celiker, Furkan Çam, Berru Yargı Özkoçak

Corresponding author:

Hande Celiker

Marmara University School of Medicine, Department of Ophthalmology, Istanbul, Turkey.

E-mail: drhandeceliker@yahoo.com

Supplementary Table S1. Preoperative and postoperative diagnosis of eyes that underwent vitreous sampling

	Postoperative diagnosis				
**Preoperative diagnosis**	**Fungal EE**	**Bacterial EE**	**PVRL**	**CMV retinitis**	**Toxocariasis**
**Unidentified uveitis (n=11)**	-	-	-	1	-
**Fungal EE (n=9)***	5	-	-	-	-
**Bacterial EE (n=10)***	-	4	-	-	-
**PVRL (n=2)**	-	-	2	-	-
**CMV retinitis (n=3)**	-	-	-	3	-
**Toxocariasis (n=1)**	-	-	-	-	-
**Total (n=36)**	5	4	2	4	-

EE, Endogenous endophthalmitis; **PVRL**, Primary vitreoretinal lymphoma; **CMV**, Cytomegalovirus.

*2 eyes with fungal EE and one eye with bacterial EE were still considered as infectious uveitis due to positive blood cultures, history of predisposing risk factors, and peroperative highly suspected retinal findings associated with EE.
