# Supplementary material for: Outcomes of pars plana vitrectomy in the management and diagnosis of patients with infectious, non-infectious, and unidentified uveitis
Source: Graefes Arch Clin Exp Ophthalmol. 2024 Feb 16;262(7):2237–46. doi: 10.1007/s00417-024-06407-y (PMC11222255; doi:10.1007/s00417-024-06407-y)
Supplement: Supplementary file 2 — (PDF 198 kb) [file 417_2024_6407_MOESM2_ESM.pdf]

Article title: Outcomes of Pars Plana Vitrectomy in the Management and Diagnosis of Patients with Infectious, Non-infectious, and Unidentified Uveitis

Journal name: Graefe's Archive for Clinical and Experimental Ophthalmology

Authors:

Hande Celiker, Furkan Çam, Berru Yargı Özkoçak

Corresponding author:

Hande Celiker

Marmara University School of Medicine, Department of Ophthalmology, Istanbul, Turkey.

E-mail: drhandeceliker@yahoo.com

Supplementary Table 2. Details of surgical procedures in patients underwent pars plana vitrectomy

Parameters	All patients	Infectious uveitis	Non-infectious uveitis	Unidentified uveitis
**Vitreous sampling (Yes/No)**	36/26	17/3	2/22	18/-
**Combined Phacoemulsification (Yes/No)**	23/39	8/12	9/15	6/12
**Additional surgical procedures**				
**ERM peeling**	24	10	10	4
**ILM peeling**	5	2	3	-
**Primary retinectomy**	3	2	-	1
**Tamponade agents**				
**Silicone oil (SO)**	38	17	7	14
**Gas**	9	2	6	1
**Air**	3	1	1	1
**None**	12	-	10	2
**SO removal**				
**Yes/No**	27/11	12/5	6/1	9/5
**Mean time of SO extraction after PPV** (months)	8.91±5.25	10.00±5.65	8.16±2.56	8.33±6.42
**Combining ERM peeling during SO removal**	10	5	2	3

ERM, Epiretinal membrane; **ILM,** Internal limiting membrane; **PPV**, Pars plana vitrectomy.
