# Supplementary material for: Outcomes of pars plana vitrectomy in the management and diagnosis of patients with infectious, non-infectious, and unidentified uveitis
Source: Graefes Arch Clin Exp Ophthalmol. 2024 Feb 16;262(7):2237–46. doi: 10.1007/s00417-024-06407-y (PMC11222255; doi:10.1007/s00417-024-06407-y)
Supplement: Supplementary file 3 — (PDF 757 kb) [file 417_2024_6407_MOESM3_ESM.pdf]

Article title: Outcomes of Pars Plana Vitrectomy in the Management and Diagnosis of Patients with Infectious, Non-infectious, and Unidentified Uveitis

Journal name: Graefe's Archive for Clinical and Experimental Ophthalmology

Authors:

Hande Celiker, Furkan Çam, Berru Yargı Özkoçak

Corresponding author:

Hande Celiker

Marmara University School of Medicine, Department of Ophthalmology, Istanbul, Turkey.

E-mail: drhandeceliker@yahoo.com

Supplementary Table 3. Preoperative and postoperative prescribed drugs and doses in patients receiving immunosuppressants

Patient No	Age	Gender	Etiology of uveitis	IS at baseline	IS at postoperative 6 months	IS at postoperative 12 months	IS at final visit
1	45	M	Behçet's uveitis	ADA bi-weekly, AZA 150mg	ADA bi-weekly	ADA bi-weekly	ADA weekly
2	52	M	Behçet's uveitis	ADA bi-weekly	ADA bi-weekly	ADA bi-weekly	ADA bi-weekly
3	34	M	Unidentified	AZA 150mg, CsA 100mg	AZA 150mg	AZA 100mg	ADA bi-weekly
4	41	F	Unidentified	AZA 150mg, CsA 100mg, CS 16mg	AZA 150mg, CsA 100mg	None	None
5	59	F	Sarcoidosis	AZA 200mg	AZA 100mg	AZA 100mg	-
6	35	M	Behçet's uveitis	AZA 200mg, CS 64mg	AZA 100mg, ADA bi-weekly	AZA 100mg, ADA weekly	AZA 100mg, ADA weekly
7*	51	M	Toxoplasmosis	CS 16mg	None	-	-
8	50	F	Unidentified	CS 32mg	AZA 100mg	None	None
9	30	M	Behçet's uveitis	ADA bi-weekly, CS 48mg	ADA bi-wekkly	-	-
10	60	M	Unidentified	CS 64mg	None	None	None
11	29	M	Unidentified	AZA 200mg, CS 64mg	AZA 100mg, ADA bi-weekly	AZA 100mg, ADA bi-weekly	AZA 100mg, ADA bi-weekly
12	58	F	Behçet's uveitis	IFX 300mg/6week	IFX 300mg/6week	IFX 300mg/6week	IFX 300mg/6week
13	73	F	Spondyloarthropathy	MTX 15mg/week, CS 16mg	None	None	None
14	12	F	Pars planitis	CS 32mg	ADA bi-weekly	ADA bi-weekly	ADA bi-weekly
15	40	F	Unidentified	None	AZA 100mg	AZA 100mg	AZA 100mg
16	40	F	Crohn's disease	IFX 300mg/6week	IFX 300mg/6week	-	-

IS, Immunosuppressive; **ADA**, Adalimumab; **AZA**, Azathioprine; **CsA**, Cyclosporine; **IFX**, Infliximab; **MTX**, Methotrexate

**This patient with toxoplasmosis also received* trimethoprim/sulfamethoxazole and clindamycin along with corticosteroid therapy.
